# Variação Geográfica no Tempo Porta-Agulha em Pacientes com IAMCSST da Região Metropolitana do Rio de Janeiro: Resultados da Coorte EQUITY-MI

**DOI:** 10.36660/abc.20250401

**Published:** 2026-01-26

**Authors:** Eric Costa de Almeida, Felipe Neves Albuquerque, Esmeralci Ferreira, Roberto Pozzan, Pedro Pimenta de Mello Spineti, Pâmela Sousa Monteiro, Williana Oliveira de Araújo, Rhayana Vitória da Rosa Silva, Carla Maciel Caminhas, Thales Cardoso Whately, João Gabriel Monteiro Junqueira, Denilson Campos de Albuquerque

**Affiliations:** 1 Hospital Universitário Pedro Ernesto Rio de Janeiro RJ Brasil Hospital Universitário Pedro Ernesto, Rio de Janeiro, RJ – Brasil; 2 Hospital Pró-Cardíaco Rio de Janeiro RJ Brasil Hospital Pró-Cardíaco, Rio de Janeiro, RJ – Brasil; 3 Universidade do Estado do Rio de Janeiro Rio de Janeiro RJ Brasil Universidade do Estado do Rio de Janeiro, Rio de Janeiro, RJ – Brasil; 4 Hospital Samaritano Rio de Janeiro RJ Brasil Hospital Samaritano, Rio de Janeiro, RJ – Brasil; 5 Hospital Unimed-Rio Rio de Janeiro RJ Brasil Hospital Unimed-Rio, Rio de Janeiro, RJ – Brasil

**Keywords:** Infarto do Miocárdio, Reperfusão Miocárdica, Mortalidade, Terapia Trombolítica, Política de Saúde

## Abstract

**Fundamento:**

Existe uma lacuna de informações sobre as estratégias de reperfusão utilizadas e a evolução dos pacientes com infarto agudo do miocárdio com supradesnível de segmento ST (IAMCSST) no estado do Rio de Janeiro.

**Objetivo:**

Avaliar o tempo de trombólise nas diversas regiões da capital e região metropolitana do estado do Rio de Janeiro, além do desfecho de insuficiência cardíaca pós-infarto.

**Método:**

O EQUITY-MI é um estudo de coorte prospectivo de pacientes diagnosticados com IAMCSST e referenciados para um único centro de reperfusão. As variáveis contínuas foram analisadas pelo teste de Kruskal–Wallis e as categóricas pelo qui-quadrado. Para estimar o efeito do tempo porta-agulha (TPA) foram aplicados os modelos de regressão linear generalizado com distribuição gamma e função de ligação log, além da regressão quantílica para estimar o efeito nos quantis 25%, 50% e 75%, ambos ajustados para sexo, idade, raça e escolaridade. O nível de significância utilizado foi de 0,05.

**Resultados:**

Foram incluídos 457 pacientes, com média de idade de 60,4 anos. Destes, 79% receberam trombólise, com mediana de TPA de 77 minutos, e 20,9% foram trombolisados em até 30 minutos, sem diferença estatística entre as regiões (p = 0,23). A região da Baixada Fluminense apresentou maior média ajustada de TPA (165,9 minutos), com diferença de tempo para as Zona Norte (0,61; 0,44 a 0,87; p = 0,003) e Zona Oeste (0,69; 0,51 a 0,96; p = 0,022). Também apresentou maior TPA em todos os intervalos (Q25, Q50, Q75). Após o infarto, 63,5% dos pacientes apresentaram insuficiência cardíaca.

**Conclusões:**

Os pacientes do EQUITY-MI apresentaram um atraso global no TPA, com pior desempenho sistemático na Baixada Fluminense, além de uma alta incidência de insuficiência cardíaca pós-infarto.

## Graphical abstract

**Figura Central:**
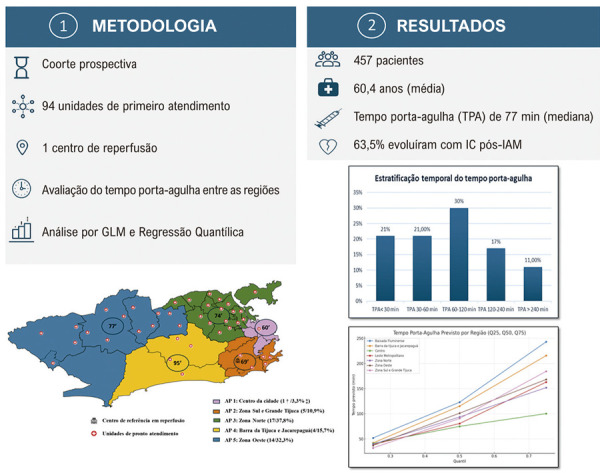
Variação Geográfica no Tempo Porta-Agulha em Pacientes com IAMCSST da Região Metropolitana do Rio de Janeiro: Resultados da Coorte EQUITY-MI

Variação geográfica no tempo porta-agulha em pacientes da região metropolitana do Rio de Janeiro: resultados do estudo EQUITY-MI. GLM: modelo de regressão linear generalizado; IAM: infarto agudo do miocárdio; IC: insuficiência cardíaca.

## Introdução

As doenças do aparelho circulatório são as principais causas de morte no Brasil. Segundo os dados de 2022 fornecidos pelo Departamento de Informática do Sistema Único de Saúde (DATASUS), o infarto agudo do miocárdio (IAM) foi a principal causa isolada de morte no país, sendo responsável por 6,34% (98.019) do total de óbitos. Já em relação ao estado do Rio de Janeiro, 85.077 pacientes foram atendidos por IAM no mesmo ano pelo Sistema Único de Saúde (SUS).

No infarto agudo do miocárdio com supradesnível do segmento ST (IAMCSST), o tempo de reperfusão e atendimento precoce exercem papel central na sobrevida dos pacientes.^1 , 2^ De fato, estudos comprovaram que a trombólise precoce,^1 , 3 - 5^ angioplastia primária^6 - 9^ e a transferência rápida para centros de referência em reperfusão associam-se à redução da mortalidade^10 - 12^ e de complicações como choque cardiogênico, insuficiência cardíaca e arritmias.^5 , 8 , 10^

No Brasil, apesar de linhas de cuidados específicas para IAM terem sido implementadas com objetivo de equalizar o acesso ao diagnóstico e tratamento precoce desses pacientes,^11 , 12^ os índices assistenciais permanecem aquém das recomendações internacionais.^13 - 15^ Em um país continental com realidades diversas como o Brasil, justificar-se-ia uma grande heterogeneidade de estratégias adotadas entre os estados.^13 , 15 , 16^ Um grande exemplo desses contrastes é o estado do Rio de Janeiro. Apesar de ser a terceira maior população do país e oitavo lugar no Índice de Desenvolvimento Humano de acordo com o Instituto Brasileiro de Geografia e Estatística (IBGE), apresenta indicadores alarmantes quanto à morbimortalidade por doenças coronarianas. O estado registrou a terceira maior taxa de mortalidade assim como a terceira maior taxa de anos de vida perdidos ajustados por incapacidade (DALY).^17 , 18^

Diante desse cenário paradoxal, o presente estudo EQUITY-MI (EQuity, Universality and Intersectionality: the challenge of ThrombolYsis in patients with Myocardial Infarction) foi desenvolvido para avaliar equidade no atendimento ao IAMCSST no Rio de Janeiro. Além de investigar a qualidade do atendimento, esse registro busca apresentar um retrato sobre a aplicação das políticas vigentes e como estas impactam no sistema de saúde ( Figura Central ).

Os objetivos do estudo EQUITY-MI foram: realizar uma análise epidemiológica de uma coorte de IAMCSST no estado do Rio de Janeiro; analisar o tempo porta-agulha (TPA) das subregiões da cidade do Rio de Janeiro e região metropolitana; e avaliar como desfecho secundário a incidência de insuficiência cardíaca pós-infarto.

## Métodos

### Desenho do estudo

Trata-se de um estudo de coorte prospectivo, realizado entre os meses de agosto de 2022 a agosto de 2024, envolvendo pacientes internados no serviço de cardiologia do Hospital Universitário Pedro Ernesto da Universidade do Estado do Rio de Janeiro (HUPE/UERJ) com diagnóstico de IAMCSST. Esta pesquisa foi conduzida pela Resolução no 466/12 do Conselho Nacional de Saúde e com a Lei nº 14.874, tendo sido aprovada pelo Comitê de Ética e Pesquisa do Hospital Universitário Pedro Ernesto sob o CAAE 62665922.2.0000.5259.

### População do estudo

**Figura 1:**
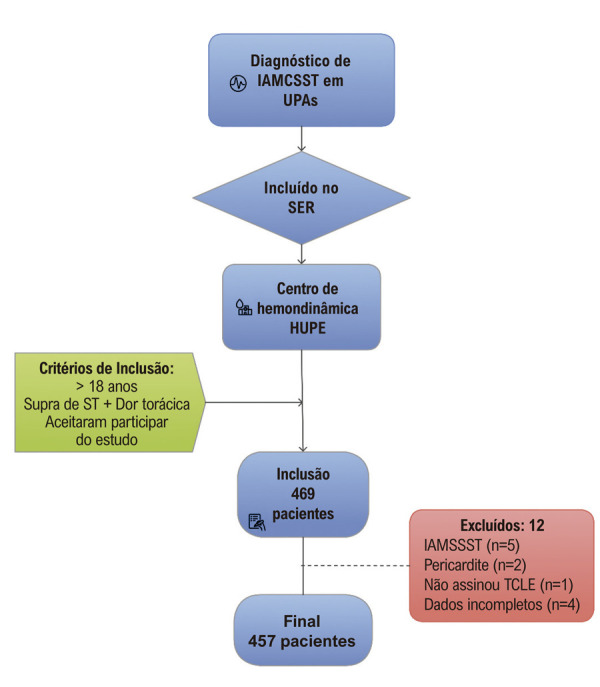
– Fluxograma de inclusão de pacientes no estudo. HUPE: Hospital Universitário Pedro Ernesto; IAMCSST: infarto agudo do miocárdio com supradesnível do segmento ST; IAMSSST: infarto agudo do miocárdio sem supradesnível do segmento ST; SER: Sistema Estadual de Regulação; TCLE: Termo de Consentimento Livre e Esclarecido; UPA: Unidade de Pronto Atendimento.

Foram encaminhados para o Setor de Hemodinâmica do HUPE/UERJ, no período de agosto de 2022 a agosto de 2024, um total de 469 pacientes com IAMCSST, de ambos os sexos, originários majoritariamente de Unidades de Pronto Atendimento (UPA) de toda a Região Metropolitana do Rio de Janeiro (Capital, Baixada Fluminense e Leste Metropolitano) via Sistema Estadual de Regulação (SER), para realização de coronariografia. Foram considerados os critérios de inclusão: pacientes > 18 anos com diagnóstico de IAMCSST e dor torácica anginosa; e encaminhamento pelas UPA/SER. Destes, 12 pacientes foram excluídos do estudo pois não assinaram o TCLE, receberam o diagnóstico de IAMCSST de forma equivocada ou apresentavam registro médico do primeiro atendimento incompleto ( Figura 1 ).

### Coleta de dados

A coleta de dados foi realizada pelos pesquisadores através de dois formulários específicos (Material Suplementar). O primeiro foi composto de informações preenchidas do hospital de origem com os seguintes itens: (i) local do atendimento; (ii) hora do início da dor torácica; (iii) hora da chegada na unidade hospitalar; (iv) realização de trombolítico; (v) data e hora da trombólise; (vi) TPA (definido como o intervalo de tempo entre a chegada na unidade hospitalar e administração do trombolítico); (vii) melhora da dor após trombólise; (viii) queda do supradesnível de segmento ST após trombólise.

O segundo formulário foi dividido em duas partes: a primeira por meio de entrevista com o paciente, e a segunda com dados clínicos obtidos no prontuário. A entrevista abordou conhecimentos sobre suas comorbidades, autodeclaração da cor da pele pela classificação do IBGE (preta, branca, parda), grau de escolaridade, renda familiar.

Na segunda parte, foram coletadas características antropométricas, classificação de Killip-Kimball para IAM na admissão^19^ e óbito hospitalar. Dentre os exames complementares, foram avaliados a coronariografia (vaso culpado, número de lesões, definição do tratamento) e o ecocardiograma (função global do ventrículo esquerdo e função do ventrículo direito).

Ambos os formulários se basearam no questionário NCDR ACTION-GWTG,^20^ o maior registro contínuo de qualidade de assistência ao infarto agudo dos Estados Unidos. Os dados foram catalogados e gerenciados no software REDCap,^21 , 22^ hospedado na plataforma da Faculdade de Ciências Médicas/Universidade do Estado do Rio de Janeiro.

### Análise estatística

As análises foram realizadas utilizando o software R (versão 4.4.1, R Foundation for Statistical Computing, Viena, Áustria) e jamovi (versão 2.6.26). O desfecho primário foi o TPA, definido como o intervalo, em minutos, entre a chegada do paciente ao hospital e a administração do agente trombolítico, mensurado exclusivamente durante o atendimento intra-hospitalar. As variáveis independentes incluíram: região de atendimento (Zona Sul/Grande Tijuca, Centro, Barra da Tijuca, Zona Norte, Zona Oeste, Baixada Fluminense e Leste Metropolitano), sexo, idade, raça/cor e escolaridade.

O TPA foi descrito por mediana e intervalos interquartis devido à assimetria da distribuição, avaliada por inspeção gráfica e testes de normalidade (Shapiro–Wilk). As variáveis categóricas foram apresentadas como frequências absolutas e percentuais. A comparação do TPA entre as diferentes regiões foi conduzida por meio do teste de Kruskal–Wallis. Para variáveis categóricas (sexo, raça e escolaridade), utilizou-se o teste do qui-quadrado. O nível de significância utilizado foi de 0,05.

Foi utilizado também como método de avaliação das diferenças regionais do TPA um modelo de regressão linear generalizado (GLM) com distribuição gamma e função de ligação log no jamovi (módulo GAMLj). A exposição principal foi regiões administrativas, com ajuste para sexo, idade (apresentada como média ± desvio-padrão), raça/cor e escolaridade. O GLM foi estimado com parâmetro de escala livre (estimado pelo modelo), e as incertezas foram obtidas por intervalos de confiança (IC) de 95% via bootstrap BCa com 2.000 reamostragens (procedimento padrão do GAMLj para GLM), complementados por testes de Wald para os efeitos principais. Para interpretação clínica, derivamos médias ajustadas do TPA por região (estimativas marginais na escala da resposta, em minutos) e razões de tempo, calculadas como exp(β), comparando cada região à referência. Como teste post hoc foi utilizada a correção de Bonferroni.

Diante da assimetria e heterocedasticidade do TPA, foi aplicada também regressão quantílica (pacote quantreg no R) para estimar o efeito das variáveis independentes nos quantis (Q25, Q50 [mediana] e Q75) da distribuição. Os coeficientes foram interpretados como diferença absoluta (em minutos) no TPA no quartil específico, comparando cada categoria à referência, com respectivos IC de 95% e valores de p.

## Resultados

### Perfil sociodemográfico

Foram avaliados 457 pacientes, com idade média de 60,4 anos, e uma maioria de pacientes do sexo masculino (72,7%). Quanto ao perfil sociodemográfico a maioria dos pacientes atendidos se autodeclaravam negros (50,7% pardos e 17,9% pretos), possuíam ensino médio (38,2%) e renda familiar entre um e dois salários-mínimos (35,8%). Na avaliação das comorbidades, destaca-se uma elevada prevalência de hipertensão arterial (64,7%), diabetes (32%) e tabagismo (34,4%).

As regiões administrativas (ou áreas de planejamento) da cidade do Rio de Janeiro foram agrupadas nas cinco principais zonas (Zona Norte e Ilha do Governador; Zona Sul e Grande Tijuca; Zona Oeste; Barra da Tijuca/Jacarepaguá; e Centro), além da Baixada Fluminense e Leste Metropolitano, macrorregiões estaduais que juntas compõem com a cidade do Rio de Janeiro a Região Metropolitana. As características sociodemográficas e clínicas (sexo, raça/cor, escolaridade, renda e comorbidades) por região encontram-se detalhadas na Tabela 1 .

**Tabela 1:** – Perfil sociodemográfico e fatores de risco cardiovasculares

	Áreas de planejamento da capital					Regiões administrativas estaduais		
	Barra da Tijuca e Jacarepaguá, n(%)	Centro, n(%)	Zona Norte, n(%)	Zona Oeste, n(%)	Zona Sul e Grande Tijuca, n(%)	Baixada Fluminense, n(%)	Leste Metropolitano, n(%)	p
**N**	50(11%)	11(2,4%)	123(27%)	107(23,4%)	35(7,6%)	95(20,7%)	36(7,8%)	
Idade (anos)*	59,8 ± 13,42	64,5 ± 10,1	59,5 ± 10,3	60,8 ± 10,9	61,0 ± 10,6	61,4 ± 9,4	59,1 ± 10,3	
**Tempo porta-agulha (min)**	95,5	60	74	77	69	109	55	0,235^†^
**Sexo feminino ^**‡**^**	11 (22,0%)	5 (45,5%)	27 (22,0%)	37 (34,6%)	10 (28,6%)	25 (26,3%)	10 (27,8%)	0,3
**Raça/cor ^**‡**^**								**0,456**
Branco	18 (36,7%)	2 (18,2%)	41 (33,3%)	30 (28,0%)	16 (45,7%)	25 (26,6%)	10 (27,8%)	
Pardo	21 (42,9%)	5 (45,5%)	58 (47,2%)	61 (57,0%)	13 (37,1%)	54 (57,4%)	19 (52,8%)	
Preto	10 (20,4%)	4 (36,4%)	24 (19,5%)	16 (15,0%)	6 (17,1%)	15 (16,0%)	7 (19,4%)	
**Grau de escolaridade ^**‡**^**								**0,074**
Analfabeto	2 (4,0%)	0 (0,0%)	5 (4,1%)	3 (2,8%)	1 (2,9%)	1 (1,1%)	2 (5,6%)	
Até o 4º ano	9 (18,0%)	2 (18,2%)	20 (16,3%)	19 (17,9%)	2 (5,7%)	16 (16,8%)	7 (19,4%)	
Até o 9º ano	16 (32,0%)	5 (45,5%)	31 (25,2%)	33 (31,1%)	9 (25,7%)	39 (41,1%)	10 (27,8%)	
Ensino médio	16 (32,0%)	2 (18,2%)	54 (43,9%)	41 (38,7%)	12 (34,3%)	36 (37,9%)	14 (38,9%)	
Ensino superior	7 (14,0%)	2 (18,2%)	13 (10,6%)	10 (9,4%)	11 (31,4%)	3 (3,2%)	3 (8,3%)	
**Renda familiar (em salários-mínimos) ^**§ ‡**^**								**0,252**
Até um	10 (20,0%)	2 (18,2%)	15 (12,3%)	22 (20,6%)	1 (2,9%)	26 (27,7%)	8 (22,2%)	
Entre um e dois	14 (28,0%)	4 (36,4%)	46 (37,7%)	39 (36,4%)	14 (40,0%)	33 (35,1%)	12 (33,3%)	
Entre dois e quatro	15 (30,0%)	3 (27,3%)	44 (36,1%)	34 (31,8%)	11 (31,4%)	24 (25,5%)	11 (30,6%)	
Maior que quatro	11 (22,0%)	2 (18,2%)	17 (13,9%)	12 (11,2%)	9 (25,7%)	11 (11,7%)	5 (13,9%)	
**Fatores de risco ^**‡**^**								
Hipertensão arterial	30 (60,0%)	8 (72,7%)	81 (65,9%)	67 (62,6%)	22 (64,7%)	61 (64,9%)	27 (75,0%)	**0,842**
Diabetes tipo 2	16 (32,7%)	2 (18,2%)	33 (27,3%)	37 (35,2%)	13 (38,2%)	31 (33,3%)	12 (35,3%)	**0,737**
Dislipidemia	3 (6,1%)	0 (0,0%)	12 (9,8%)	12 (11,8%)	3 (8,8%)	9 (9,8%)	5 (14,7%)	**0,769**
Obesidade	5 (10,4%)	0 (0,0%)	20 (16,9%)	14 (13,2%)	5 (14,3%)	13 (14,3%)	6 (19,4%)	**0,721**
DAC prévia	7 (14,0%)	1 (9,1%)	9 (7,4%)	13 (12,1%)	2 (6,1%)	13 (14,0%)	5 (14,7%)	**0,636**
Tabagismo	22(44%)	4(36%)	48(39%)	33(31%)	13(37%)	25(26,5%)	12(36%)	0,384

* Média e desvio padrão; ^†^ teste de Kruskal–Wallis; ^‡^ teste qui-quadrado; ^§^ salário-mínimo: equivalente a R$1.212,00. DAC: doença arterial coronariana. Fonte: Próprio Autor.

### Tempo porta-agulha

**Figura 2:**
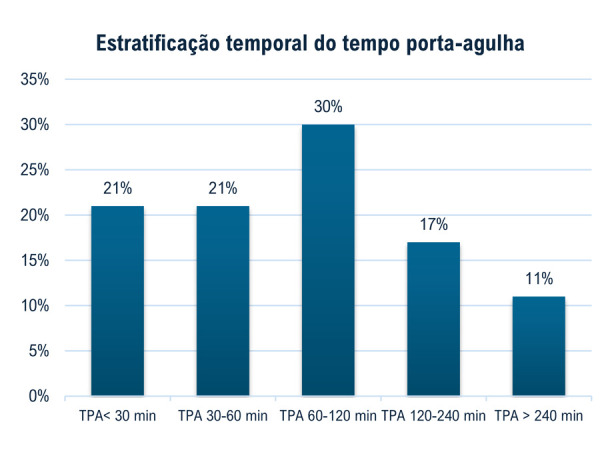
– Administração de trombolítico de acordo com o tempo porta-agulha por estratos temporais. Os estratos temporais foram: até 30 minutos; entre 30 e 60 minutos; entre 60 e 120 minutos; entre 120 e 240 minutos; maior que 240 minutos. TPA: tempo porta-agulha.

Na avaliação inicial de estratégia de reperfusão, 79% (n = 363) dos pacientes receberam trombólise, enquanto 18,5% (n = 85) não receberam e 2,4% (n = 11) não tiveram registro de trombólise informado. Dos 363 que receberam trombólise, 327 possuíam preenchimento adequado do TPA, objetivo principal do estudo, no qual o tempo mediano foi de 77 minutos, e 20,9% receberam trombólise em até 30 minutos ( Figura 2 ). O teste estatístico empregado para verificação da normalidade dos dados foi o de Shapiro–Wilk, que indicou ausência de normalidade; consequentemente, foram utilizados testes não-paramétricos.

Na comparação categórica entre regiões, o teste do qui-quadrado não evidenciou associação estatisticamente significativa (χ^2^ = 33,608; p = 0,092; N = 327).

### Distribuição geográfica das unidades de atendimento

**Figura 3:**
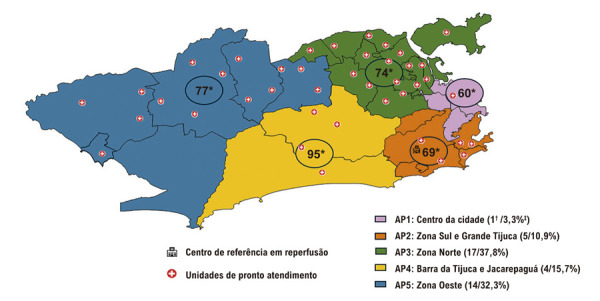
– Distribuição das Unidades de Pronto Atendimento na cidade do Rio de Janeiro e tempo mediano de porta-agulha. AP: área de planejamento. * Tempo porta-agulha em minutos; † número de UPAs de cada região; ‡ Percentual correspondente de pacientes de cada região em relação a capital. Fonte: O autor, 2025.

A Zona Norte incluiu o maior número de pacientes com 26,9% (n = 123), seguido da Zona Oeste com 23,4% (n=107) e da Baixada Fluminense com 20,7% (n = 95). Analisando o TPA como variável contínua, o teste de Kruskal–Wallis não mostrou diferença global (p = 0,235) ( Figura 3 ).

**Figura 4:**
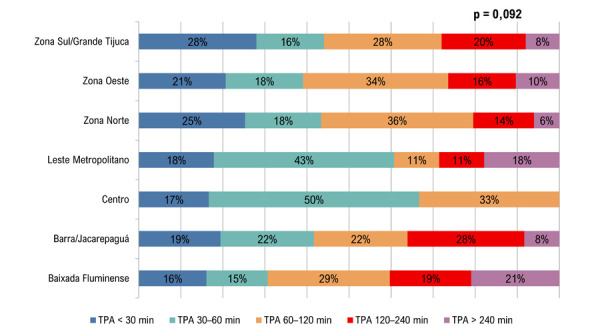
– Distribuição percentual do tempo porta-agulha por região. TPA: tempo porta-agulha.

A comparação da distribuição regional por estratos de TPA por meio do teste do qui-quadrado mostrou ausência de associação estatisticamente significativa: χ^2^(24) = 33,608; p = 0,092; N = 327. A magnitude do efeito (Cramér’s V) foi 0,156, sugerindo associação fraca entre TPA e regiões de atendimento ( Figura 4 ).

Para o desfecho TPA, empregou-se GLM com distribuição gamma e link log, incluindo regiões (exposição principal) ajustadas por sexo, idade, raça/cor e escolaridade (N = 324; IC por bootstrap BCa, 2.000 reamostragens). O modelo convergiu e apresentou desvio/graus de liberdade = 0,93. O efeito global foi limítrofe para regiões (χ^2^ = 11,78; graus de liberdade = 6; p = 0,067) e significativo para idade (χ^2^ = 4,14; p = 0,042), sem associações para sexo, raça/cor e escolaridade (todos p > 0,30). Médias ajustadas do TPA (em minutos) por região foram: Baixada Fluminense 165,9, Barra/Jacarepaguá 124,2, Centro 67,7, Leste Metropolitano 123,5, Zona Norte 100,4, Zona Oeste 114,0, Zona Sul/Grande Tijuca 112,8.

Comparadas à Baixada (referência), as razões de tempo, calculadas como exp(β), indicaram reduções na região do Centro (razão = 0,41; IC 95% 0,28 a 0,63; p = 0,031), Zona Norte (0,61; 0,44 a 0,87; p = 0,003) e Zona Oeste (0,69; 0,51 a 0,96; p = 0,022). Barra/Jacarepaguá, Leste Metropolitano e Zona Sul/Grande Tijuca apresentaram reduções numéricas de 25% a 32%, porém não significativas (p ≥ 0,10) ( Tabela 2 ). Foi realizado como teste post hoc a correção de Bonferroni com resultados conservadores.

**Tabela 2:** – Contrastes por região (GLM gamma-log) no tempo porta-agulha: razões de tempo ajustado, tomando a Baixada Fluminense como referência

Efeito	β*	exp(β)	SE^**†**^	IC95% de exp(β)		z ^**‡**^	p
Barra da Tijuca e Jacarepaguá	-0,2894346	0,75	0,2068242	0,51	1,13	-1.399.423	0,162
Centro	-0,8955465	0,41	0,415255	0,28	0,63	-2.156.618	**0,031**
Leste Metropolitano	-0,2951281	0,74	0,2220535	0,45	1,19	-1.329.085	0,184
Zona Norte	-0,5016368	0,61	0,1660576	0,44	0,87	-3.020.861	**0,003**
Zona Oeste	-0,3751464	0,69	0,1632426	0,51	0,96	-2.298.091	**0,022**
Zona Sul e Grande Tijuca	-0,3855356	0,68	0,2386339	0,41	1,11	-1.615.595	0,106

* Coeficientes (β) na escala log; ^†^ seus erros-padrão (SE); ^‡^ estatística de Wald (z); p valor e intervalo de confiança (IC) de 95% de β. O modelo foi ajustado por sexo, idade, raça/cor e escolaridade. Fonte: Próprio Autor.

**Figura 5:**
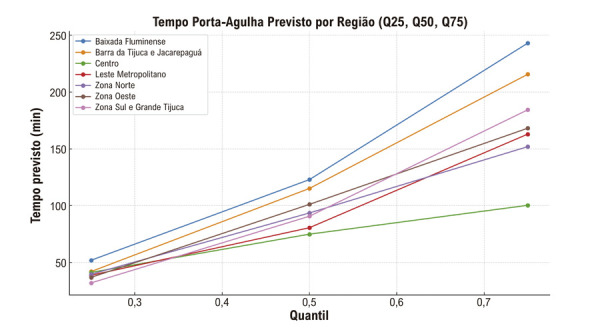
– Quantis ajustados do tempo porta-agulha (TPA) por região administrativa — regressão quantílica nos quantis 0,25, 0,50 (mediana) e 0,75 com rearranjo monotônico para evitar cruzamento de curvas e ajustado por sexo, raça/cor, escolaridade e renda.

Dada a natureza assimétrica do TPA, optou-se como teste adicional pela regressão quantílica, com objetivo de avaliar além do tempo mediano, os menores e maiores tempos de cada região. Foram estimadas regressões quantílicas nos quantis Q25, Q50 e Q75 ajustadas por região, sexo, idade, raça e escolaridade. Aplicou-se rearranjo monotônico para garantir Q25 ≤ Q50 ≤ Q75. Não foram identificadas associações estatisticamente significativas entre os preditores e o TPA nos quartis de 25%, 50% (mediana) e 75% (p > 0,05 para todas as comparações). Observou-se, contudo, tendência de tempos menores em determinadas regiões e grupos: no quantil de 25% (Q25), que representa os atendimentos mais rápidos, os tempos previstos variaram de aproximadamente 32,0 minutos na Zona Sul e Grande Tijuca a 52,0 minutos na Baixada Fluminense; na mediana (Q50), a variação foi de 74,9 minutos no Centro a 122,9 minutos na Baixada Fluminense, enquanto no quantil de 75% (Q75), que representa os 25% mais lentos, os tempos oscilaram entre 100,3 minutos no Centro e 243,1 minutos na Baixada Fluminense ( Figura 5 ).

### Complicações do infarto agudo do miocárdio

Do total de pacientes com infarto, 63,5% apresentaram algum tipo de disfunção ventricular na alta hospitalar. Apesar de não apresentar significância estatística (p = 0,10), a região da Barra da Tijuca e Jacarepaguá apresentou uma tendência de maior percentual de pacientes com disfunção grave ( Tabela 3 ).

**Tabela 3:** – Frequência de disfunção ventricular em pacientes com infarto agudo na Região Metropolitana do Rio de Janeiro

	Áreas de planejamento da capital					Regiões administrativas estaduais			
Disfunção sistólica do VE	Barra da Tijuca e Jacarepaguá	Centro	Zona Norte	Zona Oeste	Zona Sul e Grande Tijuca	Baixada Fluminense	Leste Metropolitano	Total	p*
									**0,1**
n (%)	50 (11,3%)	11 (1,2%)	117 (26%)	104 (24,2%)	34 (7,7%)	90(20,4%)	35(8%)	441	
Sem disfunção	15 (30%)	2 (18,1%)	43 (36%)	39 (37,5%)	9 (26,4%)	39(43,3%)	14(40%)	161(36,5%)	
Leve	12 (24%)	2 (18,1%)	17 (14,5%)	21 (20%)	12 (35,2%)	18(20%)	10(28,5%)	92(21%)	
Moderada	10 (20%)	7 (63,6%)	38 (32,4%)	27 (25,9%)	7 (20,5%)	20(22,2%)	7(20%)	116(26,3%)	
Grave	13 (26%)	0%	19 (16,2%)	17 (16,3%)	6 (17,6%)	13(14,4%)	4(11,4%)	72(16,3%)	

* Teste de qui-quadrado. VE: ventrículo esquerdo.

## Discussão

O estudo EQUITY-MI expõe um panorama alarmante dos pacientes com IAMCSST no estado do Rio de Janeiro. Os resultados apresentam uma população mais jovem afetada pelo IAMCSST em relação a outros países,^23 , 24^ com uma idade média de 60,4 anos, e um percentual maior da população negra atendida (68,1%). Estes achados são semelhantes a outros registros nacionais,^13 , 16 , 25 - 27^ além de uma alta prevalência de fatores de risco, como hipertensão arterial sistêmica, diabetes mellitus 2, tabagismo e história familiar de doença arterial coronariana precoce.^26^

Observa-se que no presente estudo foi priorizada a terapia fibrinolítica como estratégia inicial de reperfusão, sendo realizada em 79% dos pacientes. Os números são muito superiores aos estudos de Alves et al., com 10,5% de fibrinólise em uma cidade no Rio Grande do Sul;^16^ o VICTIM, com apenas 2,6% dos pacientes;^13^ e o registro nacional ACCEPT, no qual 18,09% dos pacientes foram trombolisados.^26^ Nota-se uma elevação gradual da taxa de reperfusão, compatível com estudos anteriores no qual o RESISST apresentou 40,7%,^14^ Lana et al. 56%,^25^ VICTIM 74,9%^13^ e ACCEPT 76,8%,^26^ ainda aquém dos padrões de países europeus^23^ e dos Estados Unidos.^24^

O estudo evidencia a vulnerabilidade do TPA no atendimento ao IAMCSST no Rio de Janeiro. O tempo mediano de 77 minutos, em nosso estudo EQUITY-MI, é melhor que o registro de Filgueiras et al. (178 minutos)^14^ e semelhante ao publicado por Moraes et al. (70 minutos),^15^ entretanto, é mais que o dobro do tempo apresentado por Vora et al. (34 minutos).^28^ Além do longo período, apenas 21% dos pacientes foram submetidos à fibrinólise em menos de 30 minutos, valor também semelhante a outros estudos regionais que apresentaram percentuais próximos de 15%,^15 , 25^ porém, muito inferiores aos registros de países desenvolvidos, próximos a 45%.^23 , 24^ Tais disparidades podem ser atribuídas a diversos fatores: a demora do diagnóstico, atrasos logísticos, falta de capacitação da equipe multidisciplinar e disparidades sociais no atendimento.

Esses dados reforçam a necessidade de intervenções na triagem e identificação do IAM de forma a mitigar atrasos no diagnóstico e tratamento. Diversos programas de telemedicina voltados para IAMCSST^29^ demonstraram, inclusive, aumento na proporção de pacientes que receberam terapia de reperfusão (60% contra 92%; RR:1,594; IC; p < 0,0001).^30^ Apesar da longa trajetória do Rio de Janeiro em telemedicina — iniciada em 1999 com o programa TIET^31^ — e do fato de a maioria das UPAs atualmente dispor desse suporte remoto, no presente estudo essa estratégia não se associou a melhor desempenho intra-hospitalar em termos de TPA.

Quanto aos atrasos logísticos, percebe-se uma incapacidade de seguir fluxos assistenciais já definidos^12 , 26^ para diagnóstico e administração de trombolítico de forma eficiente, assim como insegurança quanto à garantia de disponibilidade do fármaco em estoque e sua rápida liberação de acordo com a demanda.^32^

A falta de investimento em treinamento da equipe multidisciplinar também contribui de forma significativa para o atraso da trombólise. A capacitação permanente de médicos, enfermeiros e profissionais que realizam a triagem na emergência tem papel fundamental na redução de TPA,^26 , 33^ sendo necessária auditoria contínua dos indicadores de qualidade.^12^

A análise regional demonstrou que as zonas Norte e Oeste foram as regiões que mais incluíram pacientes neste estudo (27% e 23,4% dos pacientes, respectivamente), e com o maior número de UPA participantes (Zona Norte com 17 e Zona Oeste com 14). Esses dados são compatíveis com a importância estratégica dessas duas regiões, visto que a Zona Norte possui a maior população da cidade (cerca de 2,4 milhões de habitantes) e a Zona Oeste possui a maior mortalidade por doença isquêmica do coração (89,71/100.000 habitantes).^34^ O modelo de regressão por GLM demonstrou um padrão consistente da região da Baixada Fluminense com os maiores tempos ajustados e com diferença significativa quando comparada às regiões do Centro (~0,41×; p = 0,031), Zona Norte (~0,61×; p = 0,003) e Zona Oeste (~0,69×; p = 0,022), enquanto a regressão quantílica revelou discrepâncias que começam modestas no Q25 (Δ ≈ 20 min), tornam-se relevantes na mediana (Δ ≈ 48 min) e marcantes no Q75 (Δ ≈ 143 min), com pior desempenho sistemático também da Baixada Fluminense e desempenho consistentemente melhor da Zona Sul e Grande Tijuca. Esses achados sugerem que os atrasos intra-hospitalares prolongados não estão uniformemente distribuídos: concentram-se em determinadas regiões, sugerindo gargalos operacionais localizados.

Houve também uma alta incidência de insuficiência cardíaca pós-IAM (cerca de 64,6%). Esses dados reforçam o impacto do atraso na definição da terapia de reperfusão e suas repercussões, corroboradas pela elevada taxa de DALY do estado do Rio de Janeiro.^17^

### Limitações

O estudo foi conduzido por uma investigação a partir de um único centro, comprometendo a generalização de alguns resultados.

A região do Centro recebeu um tamanho pequeno de pacientes, dificultando possíveis avaliações sobre essa região.

A maioria dos hospitais que participaram como porta de entrada dos pacientes do estudo não possuem estrutura de engajamento para um registro exato dos dados clínicos e tempos de atendimento, podendo ocorrer algumas distorções no preenchimento dos formulários.

O estudo foca somente no componente intra-hospitalar do TPA. Atrasos pré-hospitalares (tempo sintomas-porta, transporte, decisão inicial) e tempo total de isquemia não foram avaliados, o que limita a compreensão do impacto clínico global.

## Conclusão

Os resultados do EQUITY-MI alertam para os elevados TPA encontrados. Também sugerem diferenças regionais de qualidade de atendimento, com uma tendência de pior desempenho sistemático na Baixada Fluminense e melhor desempenho consistente na Zona Sul e Grade Tijuca. A incidência de disfunção ventricular pós-IAM foi encontrada na maior parte dos pacientes estudados. Urge reduzir o tempo até o primeiro atendimento, o fluxo em triagem e capacitar a equipe multidisciplinar para diminuir principalmente o TPA e transferência para centros de reperfusão. A implementação de estratégias para reduzir tais barreiras de tratamento constitui condição indispensável para garantir equidade e universalidade no atendimento.
