# Supplementary material for: Variação Geográfica no Tempo Porta-Agulha em Pacientes com IAMCSST da Região Metropolitana do Rio de Janeiro: Resultados da Coorte EQUITY-MI
Source: Arq Bras Cardiol. 2026 Jan 26;122(12):e20250401. [Article in Portuguese] doi: 10.36660/abc.20250401 (PMC12978378; doi:10.36660/abc.20250401)
Supplement: Translation

# Translation: Geographic Variation in Door-to-Needle Time among Patients with STEMI from the Metropolitan Region of Rio de Janeiro: Results of the EQUITY-MI Cohort

**DOI:** 10.36660/abc.20250401i

**Authors:** Eric Costa de Almeida, Felipe Neves Albuquerque, Esmeralci Ferreira, Roberto Pozzan, Pedro Pimenta de Mello Spineti, Pâmela Sousa Monteiro, Williana Oliveira de Araújo, Rhayana Vitória da Rosa Silva, Carla Maciel Caminhas, Thales Cardoso Whately, João Gabriel Monteiro Junqueira, Denilson Campos de Albuquerque

**Affiliations:** 1 Brazil Hospital Universitário Pedro Ernesto, Rio de Janeiro, RJ – Brazil; 2 Brazil Hospital Pró-Cardíaco, Rio de Janeiro, RJ – Brazil; 3 Brazil Universidade do Estado do Rio de Janeiro, Rio de Janeiro, RJ – Brazil; 4 Brazil Hospital Samaritano, Rio de Janeiro, RJ – Brazil; 5 Brazil Hospital Unimed-Rio, Rio de Janeiro, RJ – Brazil

**Keywords:** Myocardial Infarction, Myocardial Reperfusion, Mortality, Thrombolytic Therapy, Health Policy

## Abstract

**Background:**

There is a gap in information regarding the reperfusion strategies used and the evolution of patients with ST-segment elevation myocardial infarction (STEMI) in the state of Rio de Janeiro.

**Objective:**

To assess thrombolysis time in the diverse regions of the state capital and metropolitan area of Rio de Janeiro, as well as the outcome of post-infarction heart failure.

**Methods:**

EQUITY-MI is a prospective cohort study of patients diagnosed with STEMI and referred to a single reperfusion center. Continuous variables were analyzed using the Kruskal–Wallis test, and categorical variables were analyzed using the chi-square test. In order to estimate the effect of door-to-needle time (DNT), generalized linear regression models with gamma distribution and log link function were applied, in addition to quantile regression to estimate the effect at the 25%, 50%, and 75% quantiles, both adjusted for sex, age, race, and education level. The significance level applied was 0.05.

**Results:**

The study included 457 patients, with a mean age of 60.4 years, 79% of whom received thrombolysis, with a median DNT of 77 minutes, and 20.9% were thrombolyzed within 30 minutes, with no statistical difference between regions (p = 0.23). The Baixada Fluminense region presented the highest adjusted mean DNT (165.9 minutes), with a time difference compared to the North Zone (0.61; 0.44 to 0.87; p = 0.003) and West Zone (0.69; 0.51 to 0.96; p = 0.022). It also presented the highest DNT in all intervals (Q25, Q50, Q75). After infarction, 63.5% of patients presented heart failure.

**Conclusion:**

There was a general delay in DNT for patients included in the EQUITY-MI study, with worse systematic performance in the Baixada Fluminense region, in addition to a high incidence of post-infarction heart failure.

## Introduction

Diseases of the circulatory system are the leading causes of death in Brazil. According to 2022 data provided by the Department of Informatics of the Brazilian Unified Health System (DATASUS), acute myocardial infarction (AMI) was the single leading cause of death nationwide, accounting for 6.34% (98,019) of all deaths. In relation to the state of Rio de Janeiro, 85,077 patients were treated for AMI in the same year within the Brazilian Unified Health System (SUS).

In acute myocardial infarction with ST-segment elevation (STEMI), reperfusion time and early care play a central role in patient survival.^1 , 2^Studies have shown that early thrombolysis,^1 , 3 - 5^primary angioplasty,^6 - 9^and rapid transfer to reperfusion referral centers are associated with reduced mortality^10 - 12^and complications such as cardiogenic shock, heart failure, and arrhythmias.^5 , 8 , 10^

In Brazil, even though specific care pathways for AMI have been implemented with the goal of equalizing access to early diagnosis and treatment of these patients,^11 , 12^care rates remain below international recommendations.^13 - 15^In continent-sized countries with diverse circumstances like Brazil, widespread heterogeneity of strategies adopted among states would be justified.^13 , 15 , 16^A prime example of these contrasts is the state of Rio de Janeiro. Although it has the third largest population in Brazil and ranks eighth in the Human Development Index according to the Brazilian Institute of Geography and Statistics (IBGE), it presents alarming indicators regarding morbidity and mortality from coronary diseases. The state of Rio de Janeiro recorded the third highest mortality rate, in addition to the third highest rate of disability-adjusted life years (DALYs).^17 , 18^

Faced with this paradoxical scenario, the EQUITY-MI (EQuity, Universality and Intersectionality: the challenge of ThrombolYsis in patients with Myocardial Infarction) study was developed to assess equity in care for patients with STEMI in Rio de Janeiro. In addition to investigating the quality of care, this registry sought to offer an overview of the application of current policies and how they impact the health system ( Central Illustration ).

The objectives of the EQUITY-MI study were as follows: to perform an epidemiological analysis of a cohort of patients with STEMI in the state of Rio de Janeiro; to analyze door-to-needle time (DNT) in the sub-regions of the city of Rio de Janeiro and its metropolitan area; and to assess the incidence of post-infarction heart failure as a secondary outcome.

## Methods

### Study design

This prospective cohort study, conducted between August 2022 and August 2024, included patients with STEMI diagnosis hospitalized in the cardiology service of the Pedro Ernesto University Hospital of the Rio de Janeiro State University (HUPE/UERJ). This study was conducted in accordance with resolution number 466/12 of the Brazilian National Health Council and law number 14.874, and it received approval from the Ethics and Research Committee of the Pedro Ernesto University Hospital under CAAE 62665922.2.0000.5259.

### Study population

A total of 469 patients of both sexes with STEMI were referred to the HUPE/UERJ hemodynamics sector, from August 2022 to August 2024. These patients were mainly referred from emergency care units (UPAs, acronym in Portuguese) throughout the Metropolitan Region of Rio de Janeiro (capital city, Baixada Fluminense, and Metropolitan East) via the state regulation system (SER, acronym in Portuguese), for coronary angiography. Inclusion criteria were as follows: patients > 18 years old with STEMI diagnosis and anginal chest pain; and referral from UPA/SER. Of these, 12 patients were excluded from the study for the following reasons: they did not sign the informed consent form; they received an erroneous diagnosis of STEMI; or their initial medical care records were incomplete ( Figure 1 ).

**Figure 1:** – Patient inclusion flowchart. FICF: free and informed consent form; HUPE: Pedro Ernesto University Hospital; NSTEMI: non-ST-segment elevation myocardial infarction; STEMI: ST-segment elevation myocardial infarction; SER: state regulation system; UPA: emergency care unit.

### Data collection

Data collection was carried out by the researchers using two specific forms (Supplementary Material). The first consisted of information completed by the referring hospital with the following items: (i) place of care; (ii) time of onset of chest pain; (iii) time of arrival at the hospital unit; (iv) thrombolytic administration; (v) date and time of thrombolysis; (vi) DNT (defined as the time interval between arrival at the hospital unit and thrombolytic administration); (vii) improvement of pain after thrombolysis; (viii) ST-segment elevation after thrombolysis.

The second form was divided into two parts. The first was applied by means of an interview with the patient, and the second comprised clinical data obtained from the medical record. The interview addressed knowledge about their comorbidities, self-declaration of skin color according to the classification used by the IBGE (Black, White, or Pardo), education level, and family income.

In the second part, anthropometric characteristics, Killip-Kimball classification of AMI at admission,^19^and hospital death were collected. The complementary exams included coronary angiography (culprit vessel, number of lesions, treatment definition) and echocardiography (global left ventricular function and right ventricular function).

Both forms were based on the NCDR ACTION-GWTG questionnaire,^20^the largest continuous registry of quality of AMI care in the United States. The data were cataloged and managed using REDCap software,^21 , 22^hosted on the platform of the Faculty of Medical Sciences, Rio de Janeiro State University.

### Statistical analysis

Analyses were performed using R software (version 4.4.1, R Foundation for Statistical Computing, Vienna, Austria) and jamovi (version 2.6.26). The primary outcome was DNT, defined as the interval, in minutes, between the patient’s arrival at the hospital and the administration of the thrombolytic agent, measured exclusively during in-hospital care. Independent variables included: region where care was provided (South Zone/Grande Tijuca, Center, Barra da Tijuca, North Zone, West Zone, Baixada Fluminense, and Metropolitan East), sex, age, race/color, and education level.

DNT was described by median and interquartile ranges due to asymmetrical distribution, as assessed by graphical inspection and normality tests (Shapiro–Wilk). Categorical variables were presented as absolute and percentage frequencies. The comparison of DNT between different regions was conducted with the Kruskal–Wallis test. For categorical variables (sex, race, and education), the chi-square test was used. The significance level was set at 0.05.

A generalized linear regression model (GLM) with gamma distribution and log link function in jamovi (GAMLj module) was also used as a method to assess regional differences in DNT. The main exposure was administrative regions, adjusted for sex, age (presented as mean ± standard deviation), race/color, and education. GLM was estimated with a free-scale parameter (estimated by the model), and uncertainties were obtained by 95% confidence intervals (CI) via bootstrap BCa with 2,000 resamplings (standard procedure of GAMLj for GLM), complemented by Wald tests for main effects. For clinical interpretation, we derived adjusted mean DNT by region (marginal estimates on the response scale, in minutes) and time ratios, calculated as exp(β), comparing each region to the reference. Bonferroni correction was used as a post hoc test.

Given the asymmetry and heteroscedasticity of DNT, quantile regression (quantreg package in R) was also applied to estimate the effect of the independent variables on the quantiles (Q25, Q50 [median], and Q75) of distribution. Coefficients were interpreted as the absolute difference (in minutes) in DNT in the specific quartile, comparing each category to the reference, with respective 95% CI and p values.

## Results

### Sociodemographic profile

This study assessed data from 457 patients, whose mean age was 60.4 years. The majority of patients were male (72.7%). Regarding sociodemographic profile, 50.7% of patients self-identified as Pardo, and 17.9% self-identified as Black; 38.2% of patients had completed secondary education; and 35.8% had a family income between one and two times minimum wage. In the assessment of comorbidities, a high prevalence of hypertension (64.7%), diabetes (32%), and smoking (34.4%) stood out.

The administrative regions (or planning areas) of the city of Rio de Janeiro were grouped into five main zones (North Zone and Ilha do Governador; South Zone and Grande Tijuca; West Zone; Barra da Tijuca/Jacarepaguá; and Center), in addition to the Baixada Fluminense and Metropolitan East, which are macro-regions of the state of Rio de Janeiro that, together with the city of Rio de Janeiro, make up the Metropolitan Region. The sociodemographic and clinical characteristics (sex, race/color, education, income, and comorbidities) by region are detailed in Table 1 .

**Table1:** – Sociodemographic profile and cardiovascular risk factors

	Planning areas of the capital city					Administrative regions of the state of Rio de Janeiro		
	Barra da Tijuca and Jacarepaguá, n(%)	Center, n(%)	North Zone, n(%)	West Zone, n(%)	South Zone and Grande Tijuca, n(%)	Baixada Fluminense, n(%)	Metropolitan East, n(%)	p
**N**	50(11%)	11(2.4%)	123(27%)	107(23.4%)	35(7.6%)	95(20.7%)	36(7.8%)	
Age (years)*	59.8 ± 13.42	64.5 ± 10.1	59.5 ± 10.3	60.8 ± 10.9	61.0 ± 10.6	61.4 ± 9.4	59.1 ± 10.3	
**Door-to-needle time (min)**	95.5	60	74	77	69	109	55	0.235^†^
**Female sex^**‡**^**	11 (22.0%)	5 (45.5%)	27 (22.0%)	37 (34.6%)	10 (28.6%)	25 (26.3%)	10 (27.8%)	0.3
**Race/skin color^**‡**^**								**0.456**
White	18 (36.7%)	2 (18.2%)	41 (33.3%)	30 (28.0%)	16 (45.7%)	25 (26.6%)	10 (27.8%)	
Pardo	21 (42.9%)	5 (45.5%)	58 (47.2%)	61 (57.0%)	13 (37.1%)	54 (57.4%)	19 (52.8%)	
Black	10 (20.4%)	4 (36.4%)	24 (19.5%)	16 (15.0%)	6 (17.1%)	15 (16.0%)	7 (19.4%)	
**Education level^**‡**^**								**0.074**
Illiterate	2 (4.0%)	0 (0.0%)	5 (4.1%)	3 (2.8%)	1 (2.9%)	1 (1.1%)	2 (5.6%)	
Up to fourth grade	9 (18.0%)	2 (18.2%)	20 (16.3%)	19 (17.9%)	2 (5.7%)	16 (16.8%)	7 (19.4%)	
Up to ninth grade	16 (32.0%)	5 (45.5%)	31 (25.2%)	33 (31.1%)	9 (25.7%)	39 (41.1%)	10 (27.8%)	
Secondary education	16 (32.0%)	2 (18.2%)	54 (43.9%)	41 (38.7%)	12 (34.3%)	36 (37.9%)	14 (38.9%)	
Tertiary education	7 (14.0%)	2 (18.2%)	13 (10.6%)	10 (9.4%)	11 (31.4%)	3 (3.2%)	3 (8.3%)	
**Family income (times minimum wage)^**§‡**^**								**0.252**
Up to one	10 (20.0%)	2 (18.2%)	15 (12.3%)	22 (20.6%)	1 (2.9%)	26 (27.7%)	8 (22.2%)	
Between one and two	14 (28.0%)	4 (36.4%)	46 (37.7%)	39 (36.4%)	14 (40.0%)	33 (35.1%)	12 (33.3%)	
Between two and four	15 (30.0%)	3 (27.3%)	44 (36.1%)	34 (31.8%)	11 (31.4%)	24 (25.5%)	11 (30.6%)	
Greater than four	11 (22.0%)	2 (18.2%)	17 (13.9%)	12 (11.2%)	9 (25.7%)	11 (11.7%)	5 (13.9%)	
**Risk factors^**‡**^**								
Arterial hypertension	30 (60.0%)	8 (72.7%)	81 (65.9%)	67 (62.6%)	22 (64.7%)	61 (64.9%)	27 (75.0%)	**0.842**
Type 2 diabetes	16 (32.7%)	2 (18.2%)	33 (27.3%)	37 (35.2%)	13 (38.2%)	31 (33.3%)	12 (35.3%)	**0.737**
Dyslipidemia	3 (6.1%)	0 (0.0%)	12 (9.8%)	12 (11.8%)	3 (8.8%)	9 (9.8%)	5 (14.7%)	**0.769**
Obesity	5 (10.4%)	0 (0.0%)	20 (16.9%)	14 (13.2%)	5 (14.3%)	13 (14.3%)	6 (19.4%)	**0.721**
Prior CAD	7 (14.0%)	1 (9.1%)	9 (7.4%)	13 (12.1%)	2 (6.1%)	13 (14.0%)	5 (14.7%)	**0.636**
Smoking	22(44%)	4(36%)	48(39%)	33(31%)	13(37%)	25(26.5%)	12(36%)	0.384

* Mean and standard deviation;^†^Kruskal–Wallis test;^‡^chi-square test;^§^minimum wage: corresponds to 1212.00 Brazilian reals. CAD: coronary artery disease. Source: the author.

### Door-to-needle time

In the initial assessment of reperfusion strategy, 79% (n = 363) of patients received thrombolysis, whereas 18.5% (n = 85) did not, and 2.4% (n = 11) had no record of thrombolysis. Among the 363 patients who received thrombolysis, DNT (the main objective of this study) was adequately filled out for 327, with a median time of 77 minutes, and 20.9% received thrombolysis within 30 minutes ( Figure 2 ). The statistical test used to verify the normality of the data was the Shapiro-Wilk test, which indicated a lack of normality; consequently, non-parametric tests were used.

**Figure 2:** – Thrombolytic administration according to door-to-needle time by time strata. The time strata were: up to 30 minutes; between 30 and 60 minutes; between 60 and 120 minutes; between 120 and 240 minutes; more than 240 minutes. DNT: door-to-needle time.

In the categorical comparison between regions, the chi-square test did not show a statistically significant association (χ^2^= 33.608; p = 0.092; N = 327).

### Geographic distribution of care units

The North Zone included the largest number of patients (26.9%; n = 123), followed by the West Zone (23.4%; n = 107) and the Baixada Fluminense (20.7%; n = 95). When analyzing DNT as a continuous variable, the Kruskal–Wallis test showed no overall difference (p = 0.235) ( Figure 3 ).

**Figure 3:** – Distribution of emergency care units in the city of Rio de Janeiro and median door-to-needle time. AP: planning area.*Door-to-needle time in minutes; † Number of UPAs (Emergency Care Units) in each region; ‡ Corresponding percentage of patients from each region in relation to the capital.. Source: the author, 2025.

Comparison of regional distribution according to DNT strata using the chi-square test showed no statistically significant association: χ^2^(24) = 33.608; p = 0.092; N = 327. Effect magnitude (Cramér’s V) was 0.156, suggesting a weak association between DNT and care regions ( Figure 4 ).

**Figure 4:** – Percentage distribution for door-to-needle time by region. DNT: door-to-needle time.

For the outcome of DNT, GLM with gamma distribution and log link was used, including regions (main exposure) adjusted for sex, age, race/color, and education (N = 324; CI by bootstrap BCa, 2,000 resamplings). The model converged, with deviance/degrees of freedom = 0.93. The overall effect was borderline for regions (χ^2^= 11.78; degrees of freedom = 6; p = 0.067) and significant for age (χ^2^= 4.14; p = 0.042), with no associations for sex, race/color, and education (all p > 0.30). Adjusted average DNTs by region were as follows: 165.9 minutes in Baixada Fluminense, 124.2 minutes in Barra da Tijuca/Jacarepaguá, 67.7 minutes in the Center, 123.5 minutes in Metropolitan East, 100.4 minutes in the North Zone, 114.0 minutes in the West Zone, and 112.8 minutes in the South Zone/Grande Tijuca.

Compared to Baixada Fluminense (reference), time ratios, calculated as exp(β), indicated reductions in the following regions: Center (ratio = 0.41; 95% CI 0.28 to 0.63; p = 0.031), North Zone (0.61; 0.44 to 0.87; p = 0.003), and West Zone (0.69; 0.51 to 0.96; p = 0.022). Barra da Tijuca/Jacarepaguá, Metropolitan East, and South Zone/Greater Tijuca showed numerical reductions of 25% to 32%; however, they were not statistically significant (p ≥ 0.10) ( Table 2 ). Bonferroni correction was performed as a post hoc test with conservative results.

**Table 2:** – Contrasts by region (GLM gamma-log) in door-to-needle time: adjusted time ratios, taking the Baixada Fluminense as a reference

Effect	β*	exp(β)	SE^**†**^	95% CI of exp(β)		z^**‡**^	p
Barra da Tijuca and Jacarepaguá	-0.2894346	0.75	0.2068242	0.51	1.13	-1.399.423	0.162
Center	-0.8955465	0.41	0.415255	0.28	0.63	-2.156.618	**0.031**
Metropolitan East	-0.2951281	0.74	0.2220535	0.45	1.19	-1.329.085	0.184
North Zone	-0.5016368	0.61	0.1660576	0.44	0.87	-3.020.861	**0.003**
West Zone	-0.3751464	0.69	0.1632426	0.51	0.96	-2.298.091	**0.022**
South Zone and Grande Tijuca	-0.3855356	0.68	0.2386339	0.41	1.11	-1.615.595	0.106

* Coefficients (β) on the log scale;^†^their standard errors (SE);^‡^Wald statistic (z); p value and 95% confidence interval (CI) of β. The model was adjusted for sex, age, race/color, and education. Source: the author.

Given the asymmetrical nature of the DNT, quantile regression was chosen as an additional test, with the objective of assessing, in addition to median time, the shortest and longest times in each region. Quantile regressions were estimated at quantiles Q25, Q50, and Q75, adjusted for region, sex, age, race, and education level. Monotonic rearrangement was applied to ensure Q25 ≤ Q50 ≤ Q75. No statistically significant associations were identified between the predictors and DNT at the 25%, 50% (median), and 75% quartiles (p > 0.05 for all comparisons). However, a trend towards shorter times was observed in certain regions and groups. In the 25% quartile (Q25), which represents the fastest care, predicted times ranged from approximately 32.0 minutes in the South Zone and Greater Tijuca to 52.0 minutes in the Baixada Fluminense. In the median (Q50), the variation was from 74.9 minutes in the Center to 122.9 minutes in Baixada Fluminense, whereas, in the 75% quartile (Q75), which represents the 25% slowest, the times ranged from 100.3 minutes in the Center to 243.1 minutes in Baixada Fluminense ( Figure 5 ).

**Figure 5:** – Adjusted quantiles of door-to-needle time (DNT) by administrative region — quantile regression at 0.25, 0.50 (median) and 0.75 quantiles with monotonic rearrangement to avoid curve crossing and adjusted for sex, race/color, education and income.

### Acute myocardial infarction complications

Of all patients with infarction, 63.5% had some type of ventricular dysfunction upon hospital discharge. Although not statistically significant (p = 0.10), the Barra da Tijuca and Jacarepaguá region showed a trend towards a higher percentage of patients with severe dysfunction ( Table 3 ).

**Table 3:** – Frequency of ventricular dysfunction in patients with acute myocardial infarction in the Metropolitan Region of Rio de Janeiro

	Planning areas of the capital city					Administrative regions of the state of Rio de Janeiro			
LV systolic dysfunction	Barra da Tijuca and Jacarepaguá	Center	North Zone	West Zone	South Zone and Grande Tijuca	Baixada Fluminense	Metropolitan East	Total	p*
									**0.1**
n (%)	50 (11.3%)	11 (1.2%)	117 (26%)	104 (24.2%)	34 (7.7%)	90(20.4%)	35(8%)	441	
No dysfunction	15 (30%)	2 (18.1%)	43 (36%)	39 (37.5%)	9 (26.4%)	39(43.3%)	14(40%)	161(36.5%)	
Mild	12 (24%)	2 (18.1%)	17 (14.5%)	21 (20%)	12 (35.2%)	18(20%)	10(28.5%)	92(21%)	
Moderate	10 (20%)	7 (63.6%)	38 (32.4%)	27 (25.9%)	7 (20.5%)	20(22.2%)	7(20%)	116(26.3%)	
Severe	13 (26%)	0%	19 (16.2%)	17 (16.3%)	6 (17.6%)	13(14.4%)	4(11.4%)	72(16.3%)	

* Chi-square test. LV: left ventricle.

## Discussion

The EQUITY-MI study exposes an alarming panorama in relation to patients with STEMI in the state of Rio de Janeiro. The results show a younger population affected by STEMI compared to other countries,^23 , 24^with a mean age of 60.4 years, and a higher percentage of the Black and Pardo population receiving care (68.1%). These findings are similar to other national registries,^13 , 16 , 25 - 27^as is the high prevalence of risk factors, such as systemic arterial hypertension, type 2 diabetes mellitus, smoking, and family history of early coronary artery disease.^26^

The present study observed that fibrinolytic therapy was prioritized as an initial reperfusion strategy, performed in 79% of patients. The numbers are far higher than in the studies by Alves et al., with 10.5% of patients receiving fibrinolysis in a city in Rio Grande do Sul;^16^VICTIM, with only 2.6% of patients;^13^and the national ACCEPT registry, in which 18.09% of patients were thrombolyzed.^26^A gradual increase in the reperfusion rate has been observed, which is consistent with previous studies, including RESISST with 40.7%;^14^Lana et al. with 56%;^25^VICTIM with 74.9%;^13^and ACCEPT with 76.8%,^26^which are still below the standards of European countries^23^and the United States.^24^

The study highlights the vulnerability of DNT in the care of patients with STEMI in Rio de Janeiro. The median time of 77 minutes, in our EQUITY-MI study, is better than the registry by Filgueiras et al. (178 minutes)^14^and similar to that published by De Marqui Moraes et al. (70 minutes);^15^however, it is more than double the time reported by Vora et al. (34 minutes).^28^In addition to the long period, only 21% of patients underwent fibrinolysis in less than 30 minutes. This value is also similar to other regional studies that showed percentages close to 15%,^15 , 25^but much lower than registries from developed countries, which are close to 45%.^23 , 24^These disparities can be attributed to several factors: delayed diagnosis, logistical delays, lack of multidisciplinary team training, and social disparities in care.

These data reinforce the need for interventions in the triage and identification of AMI in order to mitigate delays in diagnosis and treatment. Different telemedicine programs focused on STEMI^29^have demonstrated an increase in the proportion of patients who received reperfusion therapy (60% versus 92%; RR:1.594; CI; p < 0.0001).^30^Despite Rio de Janeiro’s long history in telemedicine, which began in 1999 with the TIET program,^31^and the fact that most UPAs currently have this remote support available, in the present study, this strategy was not associated with better intra-hospital performance in terms of DNT.

Regarding logistical delays, studies have observed an inability to efficiently follow pre-defined care flows^12 , 26^for diagnosis and administration of thrombolytics, in addition to uncertainty regarding the guarantee of drug availability in stock and rapid release according to demand.^32^

Lack of investment in training multidisciplinary teams also contributes significantly to delays in thrombolysis. Ongoing training of doctors, nurses, and professionals who perform triage in emergency scenarios plays a fundamental role in reducing DNT,^26 , 33^and continuous auditing of quality indicators is necessary.^12^

Regional analysis showed that the North Zone and West Zone were the regions that included the most patients in this study (27% and 23.4% of patients, respectively), as well as the regions with the highest number of participating UPAs (North Zone with 17 and West Zone with 14). These data are consistent with the strategic importance of these two regions, given that the North Zone has the largest population in the city (approximately 2.4 million inhabitants), and the West Zone has the highest mortality rate due to ischemic heart disease (89.71/100,000 inhabitants).^34^The GLM demonstrated a consistent pattern in the Baixada Fluminense region with the longest adjusted times and a significant difference when compared to the Center (~0.41×; p = 0.031), North Zone (~0.61×; p = 0.003), and West Zone (~0.69×; p = 0.022), and quantile regression revealed discrepancies that began modestly at Q25 (Δ ≈ 20 min), became relevant at the median (Δ ≈ 48 min) and marked at Q75 (Δ ≈ 143 min), with worse systematic performance in Baixada Fluminense and consistently better performance in the South Zone and Grande Tijuca. These findings suggest that prolonged intra-hospital delays are not uniformly distributed, but rather are concentrated in certain regions, suggesting localized operational bottlenecks.

There was also a high incidence of post-AMI heart failure (approximately 64.6%). These data reinforce the impact of delays in defining reperfusion therapy and its repercussions, corroborated by the high DALY rate in the state of Rio de Janeiro.^17^

### Limitations

This study was conducted as a single-center investigation, which compromises the generalizability of some results.

The Center region received a small number of patients, making it difficult to assess the impact on this region.

The majority of hospitals that participated as entry points for patients in the study do not have the engagement frameworks for accurate recording of clinical data and care times, which may lead to some distortions in filling out forms.

The study focuses only on the intra-hospital component of DNT. Pre-hospital delays (symptom-to-door time, transport, initial decision) and total ischemia time were not evaluated, which limits understanding of the overall clinical impact

## Conclusion

The EQUITY-MI results highlight the high DNTs found. They also suggest regional differences in quality of care, with a trend towards worse systematic performance in the Baixada Fluminense and better consistent performance in the Zona Sul and Grande Tijuca. The incidence of post-MI ventricular dysfunction was found in most of the patients studied. It is urgent to reduce the time to first care, the triage flow, and to train the multidisciplinary team to mainly reduce DNT and transfer to reperfusion centers. Implementing strategies to reduce such treatment barriers is essential to ensure equity and universal access to care.
